# The Feasibility of Whole-Liver Drainage with a Novel 8 mm Fully Covered Self-Expandable Metal Stent Possessing an Ultra-Slim Introducer for Malignant Hilar Biliary Obstructions

**DOI:** 10.3390/jcm11206110

**Published:** 2022-10-17

**Authors:** Saburo Matsubara, Keito Nakagawa, Kentaro Suda, Takeshi Otsuka, Masashi Oka, Sumiko Nagoshi

**Affiliations:** Department of Gastroenterology and Hepatology, Saitama Medical Center, Saitama Medical University, Saitama 350-8550, Japan

**Keywords:** malignant hilar biliary obstruction, endoscopic biliary drainage, fully covered self-expandable metal stent

## Abstract

Background: In the case of an unresectable malignant hilar biliary obstruction (MHBO), the optimal drainage method has not yet been established. Recently, an 8 mm, fully covered, self-expandable metal stent (FCSEMS) with an ultra-slim introducer has become available. In this article, the results of whole-liver drainage tests using this novel FCSEMS for MHBO are reported. Methods: Unresectable MHBOs up to Bismuth IIIa with strictures limited to the secondary branches were eligible. The proximal end of the stent was placed in such a way as to avoid blocking the side branches, and the distal end was placed above the papilla when possible. Consecutive patients treated between April 2017 and January 2021 were retrospectively analyzed. The technical and functional success rates, rates and causes of recurrent biliary obstruction (RBO), time to RBO (TRBO), revision for RBO, and adverse events (AEs) were evaluated. Results: Eleven patients (Bismuth I/II/IIIa: 1/7/3) were enrolled. Two stents were placed in nine patients and three were placed in two patients. Both the technical and functional success rates were 100%. RBO occurred in four (36%) patients due to sludge formation. Revision was performed for three patients, with the successful removal of all stents. The median TRBO was 187 days, and no late AEs other than the RBO occurred. Regarding the distal position of the stent, the RBO rate was significantly lower (14.3% vs. 75%, *p* = 0.041) and the cumulative TRBO was significantly longer (median TRBO: not reached vs. 80 days, *p* = 0.031) in the case of the placement above the papilla than the placement across the papilla. Conclusion: For unresectable MHBOs of Bismuth I, II, and IIIa, whole-liver drainage with a novel 8 mm FCSEMS possessing an ultra-slim introducer was feasible and potentially safe, with favorable stent patency. Placement above the papilla might be preferrable to placement across the papilla.

## 1. Introduction

Endoscopic retrograde cholangiopancreatography (ERCP) is the gold standard for the drainage of unresectable malignant hilar biliary obstructions (MHBOs) [1,2,3]. Although there have been a number of studies regarding the type of stent and the drainage area, the optimal drainage method has not yet been established [4,5,6,7]. Uncovered self-expandable metal stents (UCSEMSs) have a longer patency period than plastic stents (PSs) due to their larger diameter [7,8], but recurrent biliary obstruction (RBO) can still often occur because of recent advances in chemotherapy that have prolonged survival. It is problematic that UCSEMSs cannot be removed, as this can make revision difficult when RBO occurs.

Fully covered self-expandable metal stents (FCSEMSs) are removable and potentially have a longer patency period than UCSEMSs through the prevention of tumor ingrowth, whereas their placement across the side branches may result in liver abscesses [9]. In recent years, several retrospective studies have been performed using thinner, 6 mm FCSEMSs. However, the development of liver abscesses could not be eliminated, and the patency was not satisfactory [10,11]. When FCSEMSs are placed in the hilar region, it seems that whole-liver drainage using multiple stents should be performed to avoid blocking the side branches [9]. Nevertheless, the introducer of the FCSEMSs used in these studies was 8-Fr, making it difficult to implant multiple stents, particularly more than three.

**Figure 1:**
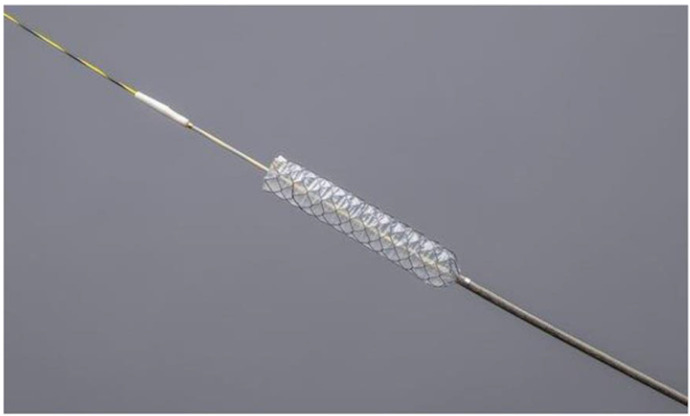
HANAROSTENT Benefit (Boston Scientific Japan, Tokyo, Japan). The fully covered, braided-type, self-expandable metal stent of 8 mm diameter with an ultra-slim introducer of 5.9-Fr. (image provided by Boston Scientific Japan).

Recently, a novel FCSEMS (HANAROSTENT Benefit; Boston Scientific Japan, Tokyo, Japan) with a 5.9-Fr ultra-slim introducer attached to a 0.025-inch guidewire was launched (Figure 1). Two introducers can be inserted into the scope channel simultaneously, facilitating the placement of multiple stents side by side. Furthermore, the stent diameter is 8 mm, which might provide a better patency than 6 mm FCSEMSs [9]. This study was conducted in order to evaluate the safety and efficacy of whole-liver drainage using this novel 8 mm FCSEMS in patients with unresectable MHBO.

## 2. Patients and Methods

### 2.1. Patients

This was a single center, retrospective cohort study using prospectively collected ERCP data, conducted at Saitama Medical Center, Saitama Medical University. We extracted data on consecutive cases, in whom whole-liver drainage was attempted using the 8 mm FCSEMS between April 2017 and January 2021. Eligibility for this procedure included unresectable MHBO of Bismuth types [12] I, II, and IIIa, in which strictures were limited to the secondary branches (the main trunk of the right anterior branch and right posterior branch). Bismuth IV and IIIa with strictures beyond the third branches were excluded because they require more than four stents for whole-liver drainage. More than four 8 mm SEMSs placed side by side could be dangerous because of the overexpansion of the bile ducts; thus, the number of stents was limited to three. In addition, Bismuth IIIb was excluded because the secondary branch of the left lobe (segment 4) is usually thin and short, making it difficult to place the 8 mm FCSEMS in the appropriate position. Patients with a surgically altered anatomy, excepting Billroth I reconstruction, were excluded. Written informed consent for the procedure was obtained from all patients prior to ERCP. The data acquisition and analysis were performed in compliance with protocols and approved by the Ethical Committee of Saitama Medical Center, Saitama Medical University (ethical approval number 2534). Informed consent for the present study was withdrawn by opting out.

### 2.2. Procedures

During ERCP, patients were sedated with midazolam and pethidine hydrochloride while in the prone position. A therapeutic duodenoscope (TJF-260V or TJF-Q290V; Olympus Medical Systems, Tokyo, Japan) with a 4.2 mm accessory channel was used for all patients. A standard ERCP catheter with a 0.025-inch guidewire (EndSelector; Boston Scientific Japan, or VisiGlide2; Olympus Medical Systems) was applied for the biliary cannulation and passage through the stricture to access the target branch. If the desired branch could not be approached, a 0.025-inch hydrophilic guidewire (Radifocus; Terumo Cop., Tokyo, Japan) was employed.

**Figure 2:**
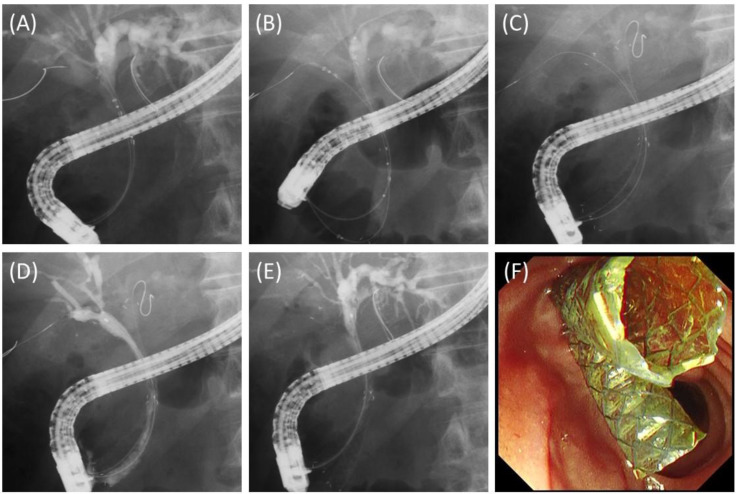
Deployment of two 8 mm FCSEMSs. (**A**) Two introducers were simultaneously inserted into the bile duct. (**B**) One stent was deployed in the left hepatic duct across the papilla. (**C**) Another stent was deployed in the right hepatic duct across the papilla. (**D**) Cholangiography after stent placement depicted all intrahepatic branches of the right hepatic lobe. (**E**) Cholangiography after stent placement depicted all intrahepatic branches of the left hepatic lobe. (**F**) Endoscopic view of two stents across the papilla.

**Figure 3:**
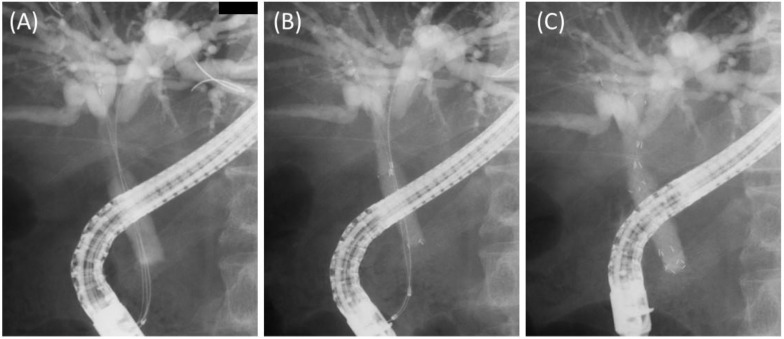
Deployment of three 8 mm FCSEMSs. (**A**) After placing three guidewires in the right anterior, right posterior, and left lateral branches, one introducer was inserted into the right posterior branch. (**B**) Following stent placement in the right posterior branch above the papilla, two introducers were inserted though the side of the first stent. (**C**) Finally, three stents were placed above the papilla without blocking the side branches.

Prior to the stent deployment, 0.025-inch guidewires were placed in all the target branches. In the case of Bismuth I or II, two introducers were inserted simultaneously into the bilateral hepatic ducts, and stents were released one by one to ensure that their proximal ends did not exceed both hepatic ducts (Figure 2). In the case of Bismuth IIIa, three stents were placed in the main trunk of the right anterior branch, the main trunk of the right posterior branch, and the left hepatic duct. For the placement of three stents, since three introducers cannot be inserted simultaneously, one stent was placed first, and then the remaining two introducers were inserted simultaneously. This is because if two stents were placed first, it would be difficult to insert the third introducer. Finally, two stents were released one by one, taking care not to block the side branches (Figure 3). The stents were placed above the papilla if the distal end of the stricture was more than 2 cm from the papilla; otherwise, the stents were placed across the papilla. Stent lengths of 60 mm, 80 mm, 100 mm, and 120 mm were available, and the appropriate length was selected for each case. In cases where the stents were placed across the papilla, endoscopic sphincterotomy was performed.

At the end of the procedure, a catheter was inserted into all the stents and contrast imaging was performed to ensure that all the branches were contrasted.

### 2.3. Outcome Measures and Definitions

We evaluated the technical success rate, functional success rate, early (up to 14 days) and late adverse events (AEs), RBO rate, causes of RBO, time to RBO (TRBO), methods and success rate of the revision for RBO, and survival. Subgroup analyses of the stent position (above or across the papilla) and timing (with or without prior RBO) were also performed in regard to the RBO. Technical success was defined as the placement of all the stents in the intended positions. AEs were defined and graded according to the lexicon of the American Society for Gastrointestinal Endoscopy [13]. A liver abscess was defined as a new fluid collection in the liver upon imaging, with fever. The definitions of the functional success rate and RBO were in accordance with the TOKYO criteria 2014 [14]. The TRBO was defined as the time from the placement of the 8 mm FCSEMSs to RBO occurrence.

### 2.4. Statistical Analyses

Descriptive continuous variables were presented as numbers (percentages) or medians (ranges). Statistical comparisons were performed with Fisher’s exact test for discrete variables and with the Mann–Whitney U test for continuous variables. The Kaplan–Meier method was used to estimate the cumulative TRBO and overall survival (OS). Deaths without RBO were treated as censored at the time of death in TRBO. The log-rank test was used to compare the TRBO in the subgroup analyses, and *p*-values of 0.05 or less were considered statistically significant. The follow-up data were gathered until November 2021. All statistical analyses were performed with EZR Ver. 1.52 (Saitama Medical Center, Jichi Medical University, Saitama, Japan) [15], which is a graphical user interface for R (the R Foundation for Statistical Computing, Vienna, Austria).

## 3. Results

### 3.1. Patient Characteristics

Eleven patients were enrolled in this study. Causes of MHBO included gallbladder cancer in four patients, bile duct cancer in four patients, pancreatic cancer in one patient, lymph node metastasis from colon cancer in one patient, and malignant lymphoma in one patient, as shown in Table 1. In terms of the Bismuth classification, type II was the most common, with seven cases, followed by three cases of type IIIa and one case of type I. Bismuth type I is a pancreatic body cancer involving the perihilar region. Four patients underwent whole-liver drainage with 8 mm FCSEMSs at index ERCP, while the other seven patients had previously undergone biliary drainage with PSs, naso-biliary tubes (NBTs), or other FCSEMSs. Of these seven patients, four had experienced RBO prior to the placement of the 8 mm FCSEMSs.

### 3.2. Details and Outcomes of the Procedures

Table 2 shows the details and outcomes of the procedures. In one case of Bismuth IIIa, cholangiography showed that communication was preserved between the right anterior and posterior branches; thus, two stents were placed in the bilateral hepatic ducts. Therefore, the placement of two stents was attempted in nine patients, and the placement of three stents was attempted in the remaining two patients, which were successful in all cases. Functional success was achieved in all cases. Stents were placed above the papilla in seven patients and across the papilla in four patients. The dilation of the stricture with an 8 mm balloon catheter was required in one patient subjected to three-stent placement. Early AEs developed in two patients with mild pancreatitis, which were resolved conventionally.

### 3.3. Long-Term Outcomes

**Figure 4:**
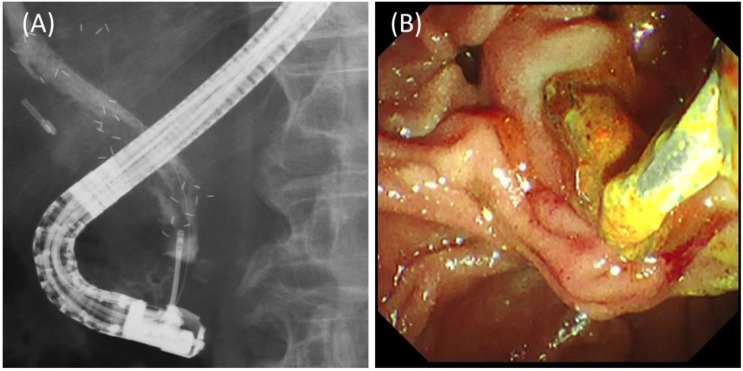
Revision for stent occlusion due to sludge in the case of three stent placements above the papilla. (**A**) Rat-tooth forceps were inserted into the bile duct and used to grasp the distal end of one stent. (**B**) Endoscopic view of the retrieval of the stent, grasped by the forceps.

Long-term outcomes of the study patients are shown in Table 3. The median follow-up period (range) was 207 days (47–378 days). RBO due to sludge occurred in four patients. Of these, revision was performed in three patients (two with across-the-papilla placement, one with above-the-papilla placement), while one patient did not undergo revision due to his poor general condition caused by the progression of the primary cancer. In the revision, all stents were successfully removed using rat-tooth forceps or a snare through the accessory channel, even in the case with above-the-papilla placement (Figure 4). The median TRBO (95% CI, confidence interval) was 187 days (49 days—NA, not applicable).

In the subgroup analyses, the RBO rate was significantly lower (14.3% vs. 75%, *p* = 0.041) and the cumulative TRBO was significantly longer (median TRBO (95% CI): NA (131 days—NA) vs. 80 days (49 days—NA), *p* = 0.031) in the case of the above-the-papilla placement than in the across-the-papilla placement. Regarding prior RBO, the RBO rate was significantly lower (14.3% vs. 75%, *p* = 0.041) and the cumulative TRBO was significantly longer (median TRBO (95% CI): NA (187 days—NA) vs. 80 days (49 days—NA), *p* = 0.005) in patients without prior RBO than in those with prior RBO. There were no late AEs other than RBO, including stent migration.

**Figure 5:**
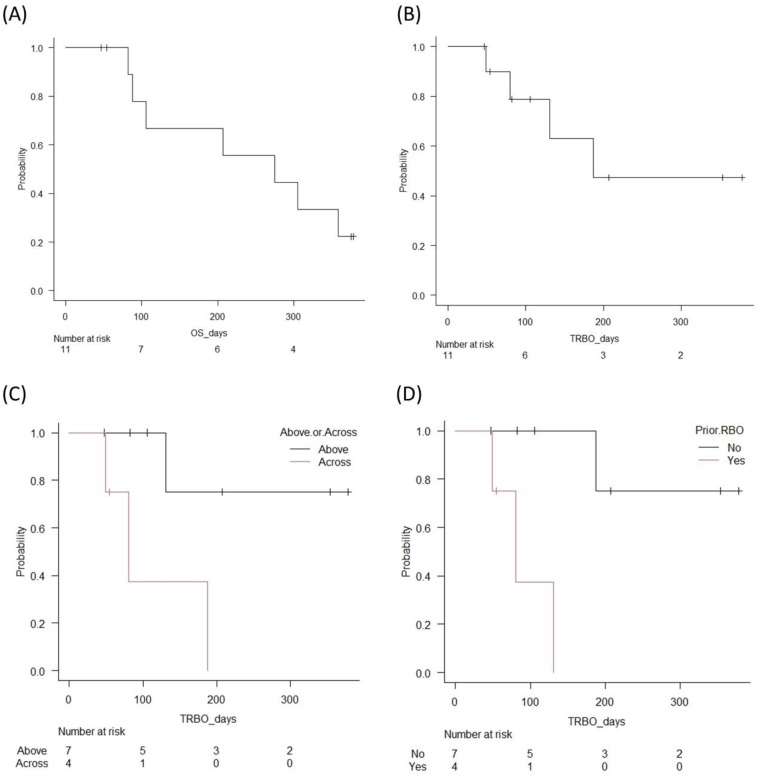
Kaplan–Meier curves for the stent patency and survival. (**A**) Overall survival (OS). Median OS was 275 days. (**B**) Time to recurrent obstruction (TRBO). Median TRBO was 187 days. (**C**) Comparison of TRBO in above and across the papilla placements. Median TRBO was significantly longer (not applicable vs. 80 days, *p* = 0.031) in above-the-papilla placement than the across-the-papilla placement. (**D**) Comparison of TRBO in cases with or without prior RBO. Median TRBO was significantly longer (not applicable vs. 80 days, *p* = 0.005) in patients without prior RBO than in those with prior RBO.

During the study period, seven patients (63.6%) died due to the progression of the primary cancer. The median OS (95% CI) was 275 days (82 days—NA). Kaplan–Meier curves for the cumulative TRBO and OS are shown in Figure 5.

## 4. Discussion

In the present study, whole-liver drainage with novel 8 mm FCSMESs for unresectable MHBOs of Bismuth type I, II, or IIIa was feasible and safe and yielded a reasonably favorable TRBO of six months. In addition, it was shown that TRBO was significantly longer in the case of the above-the-papilla placement than in the across-the-papilla placement.

In the case of palliative drainage for unresectable MHBOs, UCSMESs have been shown to have a longer patency than PSs [7,8]. In terms of the drainage area, it has been shown that drainage of more than 50% of the liver volume is associated with not only stent patency but also a better prognosis [16], and some recent studies, including an RCT, have shown that bilateral drainage is superior to unilateral drainage for stent patency [4,8]. Given recent improvements in the performance of UCSEMSs, bilateral drainage using UCSEMSs is considered the current standard of care [17,18]. However, since UCSEMSs cannot be removed, revision, with respect to multiple implanted UCSEMSs, in complex MHBO is often problematic when RBO occurs. It is also often impossible to access the undrained area where cholangitis develops. In the case of chemotherapy, it is important to avoid the interruption of chemotherapy due to RBO or cholangitis so as to maintain the treatment intensity. In addition, if conversion to surgery becomes possible after the lesion shrinks, unremovable UCSEMSs may be a hindrance to surgery. For these reasons, there are those who believe that it is better to use PSs instead of UCSEMSs for patients undergoing chemotherapy and to replace the stent periodically [19,20,21].

However, the increased number of ERCPs is problematic in terms of the patient burden and cost. Therefore, some retrospective studies were conducted using removable 6 mm FCSEMSs, expected to show a longer patency than PSs. Inoue et al. reported the application of 6 mm FCSMESs to 30 patients with Bismuth II-IV MHBO, with technical success in 28 cases (93%). One stent was placed in 10 patients and two were placed in 18 patients, with median TRBOs of 152 days and 142 days, respectively. Liver abscesses were observed in two patients (7%), and the stent was removed in one patient [10]. Yoshida et al. performed 16 procedures of 6 mm FCSMES placement in 10 patients with Bismuth II-IV MHBO, with technical success in 15 patients (94%), and two stents were placed in all the patients. The median TRBO was 113 days, and liver abscesses were observed in two patients (13%) [11]. The stent introducers used in these studies were as thick as 8-Fr, which made it impossible, in some cases, to insert a second stent even after the balloon dilation of the stricture. On the other hand, the introducer of the 8 mm FCSEMS was as thin as 5.9-Fr, facilitating successful stent placement in all patients, including three stents. The dilation of the stricture was performed in only one case. Liver abscess is a serious life-threatening AE, as well as a long-term interrupter of chemotherapy, and it should be avoided. However, even with their relatively thin diameters, the 6 mm FCSEMSs caused liver abscesses by obstructing the side branches. In the present study, no liver abscesses occurred, even though 8 mm FCSESMs were used, because they were placed without blocking the side branches. The TRBO in the present study was favorable compared to those of previous studies. The reason for this may be due to the larger diameter of the stent or the absence of Bismuth IV cases.

In bilateral drainage using UCSEMSs for MHBO, 8 mm stents are often used. Since FCSEMSs of the same diameter were used in the present study, we expected a longer patency period compared to UCSEMSs through the prevention of tumor ingrowth. However, the median TRBO for bilateral drainage using UCSEMSs in prospective studies was 200–300 days [4,7,22], which is better than the outcome of the present study. The advantage of FCSEMSs is that they prevent tumor ingrowth, but migration and sludge formation are drawbacks [23]. Because of their equal influences, the TRBOs of FCSEMSs and UCSEMSs are comparable in the case of distal MBO (DMBO) [24]. However, in MHBO, FCSEMS occlusion due to sludge is more likely to occur because of the use of longer and thinner stents than in DHBO, while ingrowth into the UCSEMs could be somewhat controlled with advances in chemotherapy, leading to a longer TRBO in the case of UCSEMSs. In fact, RBO due to sludge was much more common in the present study (36%) than that reported for FCSEMSs in DMBO. Another possible reason for the shorter TRBO in the present study, compared to that reported for UCSEMSs in MHBO, was that one-third of patients had prior RBO. In MHBO, prior cholangitis or RBO is considered a risk factor for early stent occlusion, which is thought to be due to residual sludge in the intrahepatic bile ducts [25]. In fact, patients with prior RBO had high RBO rates and shorter TRBOs than those without prior TRBO in the present study. Furthermore, the fact that one-third of the patients underwent across-the-papilla placement may also be the reason for the shorter TRBO in the present study, compared to that reported for UCSEMSs in MHBO. Above-the-papilla placement is expected to reduce the RBO by sludge formation due to less reflux of the intestinal fluid, and this possibility has been demonstrated in the case of PSs [26]. Since UCSEMSs are less likely to be occluded by sludge, there was no difference in TRBO between the placements above and across the papilla [27]. However, as FCSEMSs have a high risk of sludge formation, it is theoretically better to use above-the-papilla placement, as demonstrated in the present study. While we cannot be sure, based on the small number of cases, the TRBO of the 8 mm FCSEMSs with above-the-papilla placement might be better than that of UCSEMSs.

AEs occurred in two patients (18%) with mild pancreatitis. One patient underwent above-the-papilla placement, which was probably caused by ERCP itself. The other showed a case of two stents placed across the papilla. All four patients with across-the-papilla stenting had two stents placed, and one of them (25%) developed pancreatitis. In previous reports, in a total of 26 patients with two 6 mm CSEMSs placed across the papilla, no pancreatitis was observed [11,28]. The reason for the relatively high incidence of pancreatitis in the present study is not clear, but the difference in the diameter of stents may be the cause. In a report by Ishigaki et al., using two 8 mm UCSEMSs placed side by side across the papilla, the incidence of pancreatitis was 29%, which was similar to ours [29]. However, most of their cases were moderate, while our case was mild. High axial force is believed to be a cause of pancreatitis after SEMS [30], and the 8 mm FCSEMSs had a lower axial force than WallFlex (Boston Scientific Japan), used by Ishigaki et al., which may have resulted in the lower severity of the pancreatitis. In addition, the fact that FCSEMSs can be removed when necessary is a major advantage.

The present study has several limitations. Firstly, it was a single center, retrospective study. Therefore, selection bias and reporting bias could not be eliminated. Secondly, only a small number of cases were included, with no comparison cases. Thirdly, only cases up to Bismuth III were included, and more advanced cases were excluded. Consequently, the generalization of this method is restricted.

In conclusion, whole-liver drainage with a novel 8 mm FCSEMS possessing an ultra-slim introducer of 5.9-Fr was feasible and potentially safe and might have favorable stent patency for unresectable MHBOs of Bismuth I, II, and IIIa. Further investigations based on prospective designs involving large cohorts at multiple centers are warranted.

## Figures and Tables

**Table 1:** Patient characteristics.

Age, years	81 (60–85)
Sex, male	9 (81.8)
Primary cancer	
Gallbladder	4 (36.4)
Bile duct	4 (36.4)
Pancreas	1 (9.1)
Colon	1 (9.1)
Malignant lymphoma	1 (9.1)
Bismuth classification	
I	1 (9.1)
II	7 (63.6)
IIIa	3 (27.3)
Prior drainage	7 (63.6)
Prior RBO	4 (36.4)
Chemotherapy	6 (54.5)

Numbers are shown as numbers (%) or medians (ranges).

**Table 2:** Procedure details and outcomes.

Technical success	11 (100)
Functional success	11 (100)
CBD diameter, mm	8 (6–12)
Number of stents	
2	9 (81.8)
3	2 (18.2)
Stent length	
60 mm	12
80 mm	11
100 mm	1
Stent position	
Above the papilla	7 (63.6)
Across the papilla	4 (36.4)
Dilation of the stricture	1 (9.1)
Procedure time, mins	62 (30–84)
Early Aes	
Pancreatitis (mild)	2 (18.2)

Numbers are shown as numbers (%) or medians (ranges). CBD, common bile duct; AE, adverse event.

**Table 3:** Long-term outcomes.

Follow-up period, days	207 (47–378)
RBO	
All pts (*n* = 11)	4 (36.4)
Pts with stents above the papilla (*n* = 7)/across the papilla (*n* = 4)	1 (14.3)/3 (75)
Pts without prior RBO (*n* = 7)/with RBO (*n* = 4)	1 (14.3)/3 (75)
Causes of RBO	
Sludge	4
TRBO, days (95% CI)	
All pts (*n* = 11)	187 (49—NA)
Pts with stents above the papilla (*n* = 7)/across the papilla (*n* = 4)	NA (131—NA)/80 (49—NA)
Pts without prior RBO (*n* = 7)/with RBO (*n* = 4)	NA (187—NA)/80 (49—NA)
Revision for RBO	3 (27.3)
Exchange for 8 mm FCSEMSs	2
Exchange for NBTs	1
Late AEs other than RBO	0
OS, days (95% CI)	275 (82—NA)

Numbers are shown as numbers (%) or medians (ranges). RBO, recurrent biliary obstruction; Pt, patient; TRBO, time to recurrent biliary obstruction; NA, not applicable; FCSEMS, fully covered self-expandable metal stent; NBT, naso-biliary tube; AE, adverse event; OS, overall survival.
